# The Need for a Dynamic Approach to Health System-Centered Innovations: Comment on "What Health System Challenges Should Responsible Innovation in Health Address? Insights From an International Scoping Review"

**DOI:** 10.15171/ijhpm.2019.25

**Published:** 2019-05-26

**Authors:** Josefien van Olmen, Bruno Marchal, Button Ricarte, Wim Van Damme, Sara Van Belle

**Affiliations:** ^1^ Department of Public Health, Institute of Tropical Medicine, Antwerp, Belgium.; ^2^ Department of Primary and Interdisciplinary Care, University of Antwerp, Antwerp, Belgium.

**Keywords:** Health Systems, Health Systems Agency, Diffusion of Innovation

## Abstract

Lehoux and colleagues plea for a health systems perspective to evaluate innovations. Since many innovations and their scale-up strategies emerge from processes that are not (centrally) steered, we plea for any assessment with a dynamic, instead of a sequential, approach. We provide further guidance on how to adopt such dynamic approach, in order to better un-derstand and steer innovations for better health systems. A systems-level challenge is constituted by interactions and feedback loops between different actors and components of the health system. It is therefore essential to explore both the entry-point of innovation and the interactions with other components. If innovation is regarded as an injection of resources and opportunities into a health system, this system needs to have the capacity to transform these into desired outputs, the ‘absorption capacity.’ The highly organic diffusion of innovation in complex adapative systems cannot be easily controlled, but the system behaviours can be analysed, with occurance of phenomena such as path dependence, feedback loops, scale-free networks, emergent behaviour and phase transitions. This helps to anticipate unintended consequences, and to engage key actors in ongoing problem-solving and adaptation. By adopting a prospective approach, responsible innovation could set in motion prospective policy evaluations, which on the basis of iterative learning would allow decisionmakers to continuously adapt their policies and programmes. Priority-setting for innovation is an essentially political process that is geared towards consensus-building and grounded in values.

Innovations have driven societal progress – health systems included. Technological progress has stimulated the development of the healthcare sector, with the inventions of diagnostic tools and medical treatments. Donabedian put the spotlight on the system and processes of healthcare^1^ and since then, attention to the goals and functions of the health system started. A health system is an open and dynamic system in which “all actors, organisations, institutions and resources with the prime intent to improve health” interact with each other and with the surrounding environment.^2^ The focused attention for health systems and their goals has led to a critical review of focused interventions, and their effects on the equilibrium of health systems.

In the same line as above, Lehoux and colleagues, are critical towards innovation. They argue that not all innovation is intrinsically good and they plea for a health systems perspective to assess the need for and to evaluate innovations. In their scoping review, they have mapped health system challenges for which responsible innovation can provide answers.^3^ Their efforts are valuable to understand the largest blind spots in health systems challenges, in high-, middle-, and low-income countries. They argue that actors pursuing responsible innovation should perform a health system analysis before designing or implementing an innovation (ie, ex ante deliberation). Their mapping of needs provides the important nails for people who design hammers. However, the review ignores the fact that many innovations and their scale-up strategies emerge from processes that are not (centrally) steered. The complex nature of health systems asks for a dynamic, instead of a sequential, approach. The paper, unfortunately, does not elaborate on how the different health systems blocks interact and react to innovations, and how innovations are taken up and diffused within a health system.

In this commentary, we provide further guidance on how such dynamic approach can be assessed, in order to better understand and steer innovations for better health systems. We first discuss the methodology of the paper and then the question of how innovation in health (RIH) systems actually works. We hope to indicate how further research could be built upon this review.

## Reviewing the Methodology

One could question whether a scoping review methodology is adequate to capture societal demand for innovation. While the authors point out that a scoping review can cover literature from many disciplines, a meta-narrative review would be advisable – as was used by Greenhalgh et al on diffusion of innovation.^4^ Related to this is the choice to limit the search to the health system. A meta-narrative review would allow juxtaposing RIH with other sectors’ experience, focusing on for instance socio-technological innovation, the role of societal choices in priority-setting, etc.

For the sake of manageable data extraction, Lehoux et al kept eight components of the health system separate. While this might be necessary for keeping the review process within limits, this does not facilitate the systems approach needed to identify a ‘systems-level challenge.’ A systems-level challenge is by definition constituted by interactions and feedback loops between different actors and components of the health system. Therefore, it would have been useful to explore both the entry-point of innovation (ie, which health system components are subject to technological change and innovation) and the interactions between components of the health system (ie, undesirable and unforeseen effects) that could be triggered. The effects of the innovation in one component are indeed likely not to be contained within that component; but to be reinforced, countered or neutralized by effects in other parts of the health system. For instance, imagine that a diagnostic tool and reporting system for outbreak response is introduced, and that staff are trained accordingly. If the internet connection on which the intervention depends is too unreliable in health facilities in remote areas, then not only will the innovation fail, it will also increase inequity in service delivery. To explore such interactions, causal loop diagramming can be used to anticipate feedback loops, unforeseen desirable and non-desirable effects and the resulting emergent system behaviour.^5^ Other relevant techniques include forecasting and scenario-building.

Other assumptions the authors could have clarified include how they view the translation from demand to challenges and needs, and the assumption that frequency reflects system-level priorities (eg, reasons why there are few articles on finance as this component is very much related to factors outside the health system).

Finally, it is unclear why the authors chose to foreground the four Human Development Index (HDI) groups in their data selection. From the findings, we see that other categorizations might also be salient – for example the rural-urban divide, the mix of public-private provision, or whether there is political stability or not. It is evident that in Somalia, capacity of health stewardship is limited as it mirrors broader political unstability. The findings show that retention of health workers in rural zones across HDI groups shares more characteristics than expected. Crossing the 4 HDI with other factors would maybe have yielded less evident results.

## How Does Innovation Work in Health Systems?

The health systems dynamics framework sets out from the assumption that health systems are complex social systems, and that their state and performance is the result of the interactions between the people who are at the core of it and the dynamics between the different functions and components. The model assigns a central importance to the axis governance – health workforce-service provision – community, and the interactions between these components and with the other components.^6^ The next session provides clues for researchers to adopt a dynamic approach: (1) assess the balance between injection and the system’s capacity to absorb; (2) study the dynamics of interactions between the components; (3) focus on actors, power and regulation of power.

### Innovation as an Injection of Resources

If innovation is regarded as an injection of resources and opportunities into a health system, this system needs to have the capacity to transform these into desired outputs. Atun et al describe how this is dependant both on the innovation and the health system.^7^ In a later publication on the health systems dynamics framework, we have called this the ‘absorption capacity.’^2^ Obviously, any large external input risks to disrupt the internal system dynamics. When health systems are able to embed innovations into their organizational structure and culture, they are able to intrinsically strengthen and benefit more from additional innovations. Paradoxically, strong systems are thus easier to strengthen. Potter and Brough have provided a useful tool to assess the capacity of the system, the organisations and the actors in a health system.^8^ This implies that innovations should not only look at the mapping of health system challenges, but also at the hierarchy of needs (see below).

### Innovation From a Complexity Perspective

We now focus on the process underlying innovation. The spread of innovations can occur via diverse mechanisms, from spontaneous emergence to reengineering.^4^ In complex systems such as health systems, diffusion of innovation is usually highly organic; and an interaction between adaption of the organisation to the innovation and vice versa. Complexity theory starts with the recognition that this process cannot be easily controlled, but that we can study the behaviours of the health system in reaction to innovation, and describe phenomena such as path dependence, feedback loops, scale-free networks, emergent behaviour and phase transitions. This will help with anticipating unintended consequences that come along with innovation, and engage key actors in ongoing problem-solving and adaptation.^9^ For instance, in the scale-up of integrated care for chronic conditions such as diabetes, many health providers and businesses come up with innovations to support self-management. Digital tools are developed but their integration in physical services is piecemeal and the uptake for vulnerable people is often poor, leading to potentially more inequity. In health systems with large provider and patient freedom, such developments need steering through funding and financial protection for the poor; while in systems with a centrally governed national health system, a partnership between government and providers to improve the tool might be more useful.

### The Central Role of Health Workers and Human Capital

Any introduction of technological innovation requires a human touch and thus affects (and is affected by) the health workforce, which in turn is intricately linked to the governance component. The RIH policy process recommended by the authors^10^ would, in our view, benefit from having an associated capacity assessment and projection of how the innovation will affect, first, health worker motivation, autonomy and competences, and second, the governance component. By adopting a prospective approach, RIH could set in motion prospective policy evaluations, which on the basis of iterative learning would allow decisionmakers to continuously adapt their policies and programmes.

### Innovative for Whom?

The governance capacity is not only crucial in terms of managing the human touch in innovation, it is also central for priority-setting. Borrowing from the concept of complex adaptive governance,^11,14^ we envisage that any RIH policy process engages users, citizens, providers and policy-makers in equal measure. This raises the question of the role of the policy-maker in the RIH process and the required capabilities of policy makers.^15^ Citizen expectations will have to be managed, and this is often more difficult than expected – to be done it the right way. It is best to consider priority-setting for innovation as an essentially political process that is geared towards consensus-building and grounded in values (ie, what are we willing to spend on technological innovation X? How to ensure this is distributed equitably?). ‘Collaborative governance’ provides interesting sources for inspiration.^16-18^ Also the accountability for reasonableness framework of Norman Daniels could further feed the responsible RIH framework as proposed by the authors.^19,20^

### The Role of the State and the Capabilities of Policy-Makers

As the authors point out, the strength of government capacity is outside the remit of the paper. However, the research question touches upon the fundamental political choice of regulation of the healthcare market and state interventionism – it should be noted that Greenhalgh et al plead for tighter regulation in technologically-oriented health systems.^4^ This might not only be a choice confined to the national health system-level, but also is influenced by the competition in a global health technology market with emerging countries, such as China and India, as primary players.

## Ethical issues

Not applicable.

## Competing interests

Authors declare that they have no competing interests.

## Authors’ contributions

All authors discussed the general approach and concepts. JvO and SVB developed the outline and first draft. BM and WVD provided textual comments.

## Authors’ affiliations

^1^Department of Public Health, Institute of Tropical Medicine, Antwerp, Belgium. ^2^Department of Primary and Interdisciplinary Care, University of Antwerp, Antwerp, Belgium.
